# Supplementary material for: Role of the Anilinium Ion on the Selective Polymerization of Anilinium 2-Acrylamide-2-methyl-1-propanesulfonate
Source: Polymers (Basel). 2021 Jul 17;13(14):2349. doi: 10.3390/polym13142349 (PMC8309539; doi:10.3390/polym13142349)
Supplement: Supplementary file 1 [file polymers-13-02349-s001.zip › polymers-1277512-supplementary.pdf]

Role of the anilinium ion on the selective polymerization of anilinium 2-acrylamide-2-methyl-1-propanesulfonate

Alain S. Conejo-Dávila[1], M. Armando Moya-Quevedo[1], David Chávez-Flores[2], Alejandro Vega-Rios[1,*] and E. Armando Zaragoza-Contreras[1,*]

[1] Department of Engineering and Materials Chemistry. Centro de Investigación en Materiales Avanzados, SC. Miguel de Cervantes No. 120, Complejo Industrial Chihuahua, Chihuahua, Chih., México. C.P. 31136.
[2] Facultad de Ciencias Químicas. Universidad Autónoma de Chihuahua, Chihuahua, Chih., México. C.P. 31125.
* Correspondence: AVR alejandro.vega@cimav.edu.mx; EAZC Armando.zaragoza@cimav.edu.mx

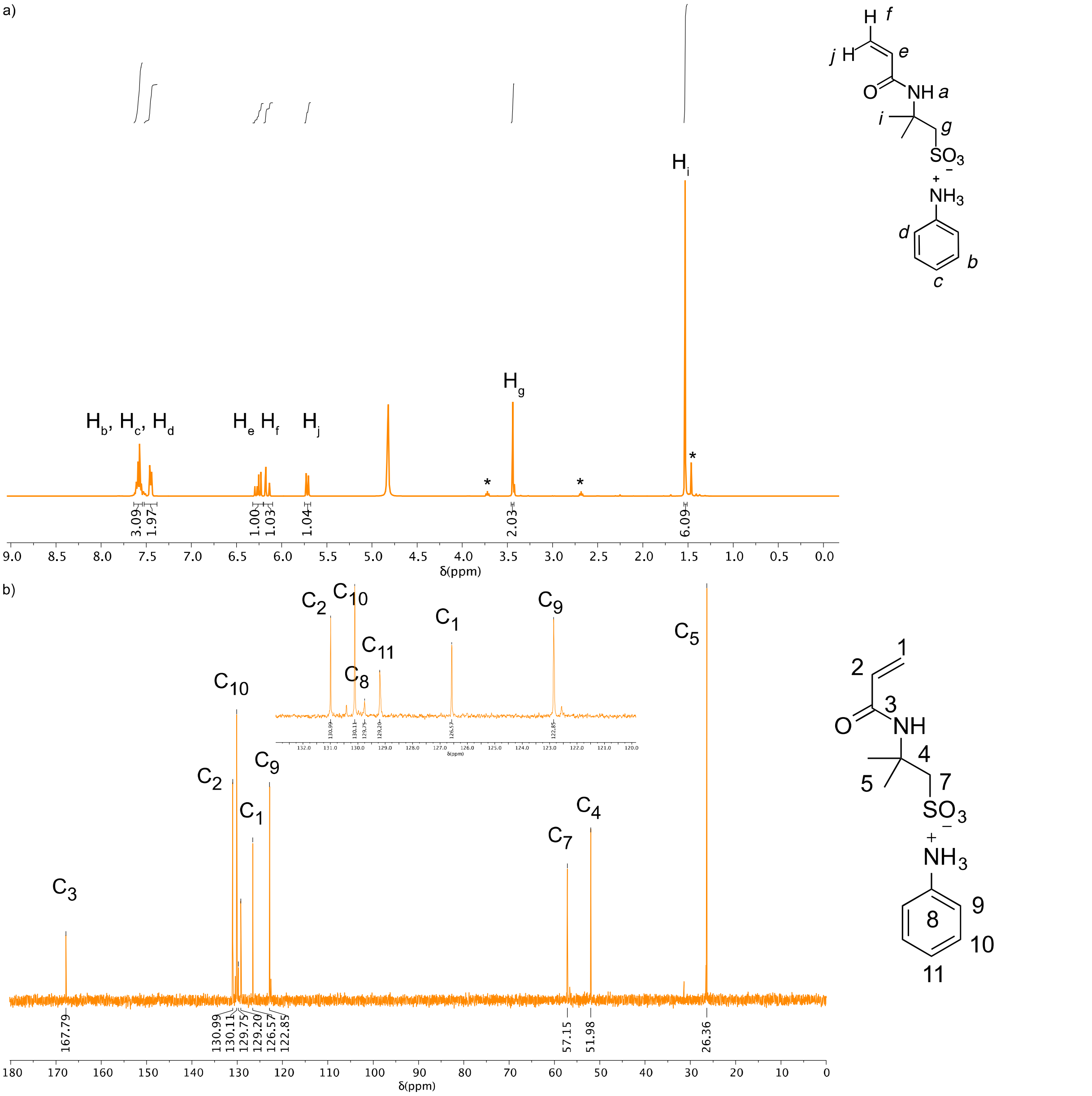

Figure S1. NMR spectra. a) ^{1}H NMR (400 MHz, Deuterium Oxide) δ 7.64 – 7.54 (m, 3H), 7.45 (dt, J = 8.0, 1.5 Hz, 2H), 6.26 (dd, J = 17.1, 10.1 Hz, 1H), 6.16 (dd, J = 17.1, 1.5 Hz, 1H), 5.72 (dd, J = 10.1, 1.5 Hz, 1H), 3.44 (s, 2H), 1.53 (s, 6H). *By-products of monomer synthesis, specifically oligomers. b) ^{13}C NMR (101 MHz, Deuterium oxide) δ 197.79, 130.99, 130.11, 129.75, 129.20, 126.57, 122.85, 57.15, 51.98, 26.36 ppm.

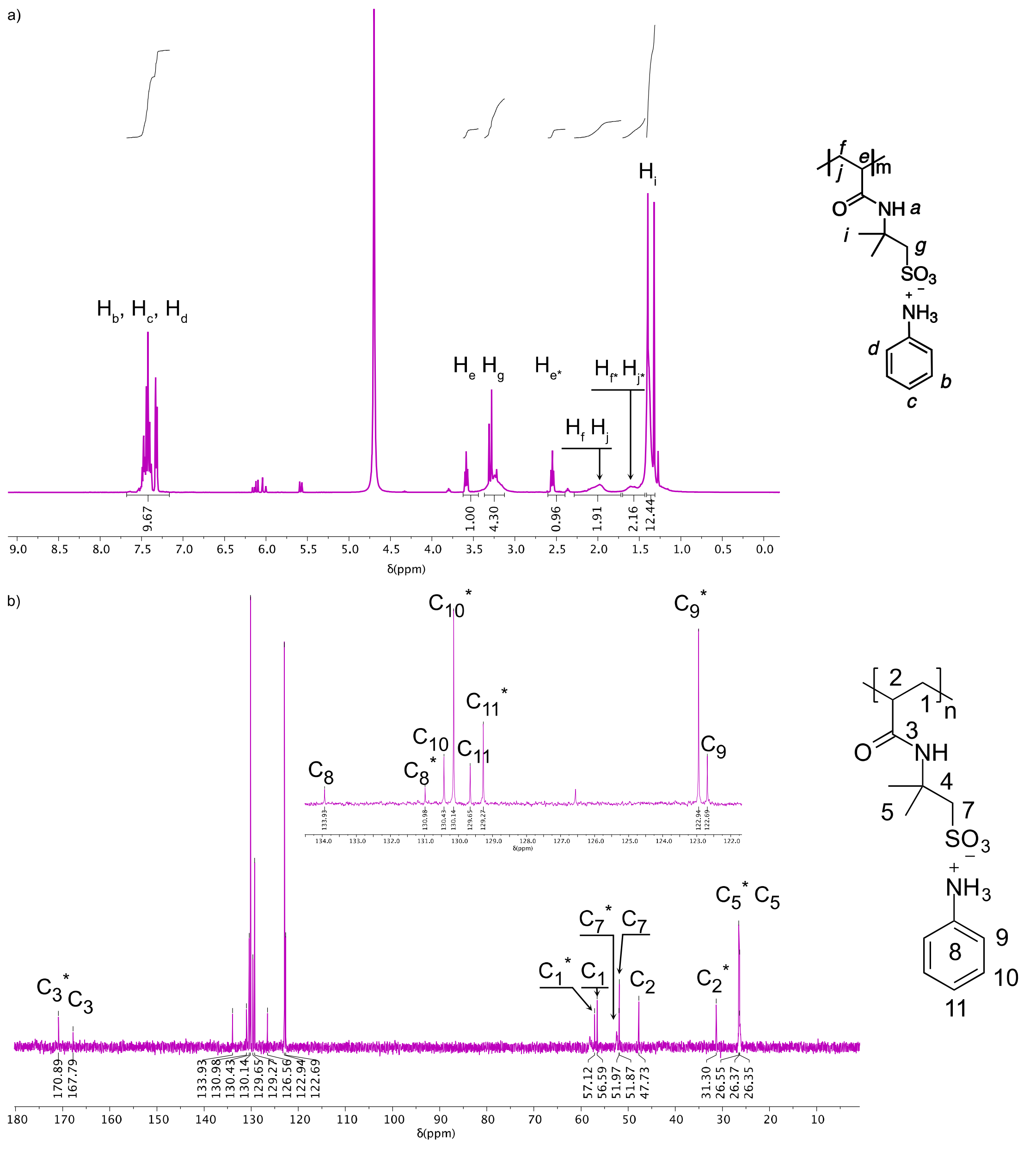

Figure S2. ^{1}H NMR (400 MHz, Deuterium Oxide) δ 7.80-7.29 (m, 10H), 3.71 (t, J = 6.3 Hz, 1H), 3.49-3.25 (m, 4H), 2.67 (t, J = 6.3Hz, 1H), 2.10 (m, 2H), 1.82-1.55 (m, 2H), 1.53-1.43 (m, 12H); ^{13}NMR (101 MHz, Deuterium Oxide) δ 170.89, 167.79, 133.93, 130.98, 130.43, 130.14, 129.65, 129.67, 126.56, 122.94, 122.69, 57.12, 56.59, 51.97, 51.87, 47.73, 31.30, 26.55, 26.37, 26.35 ppm.

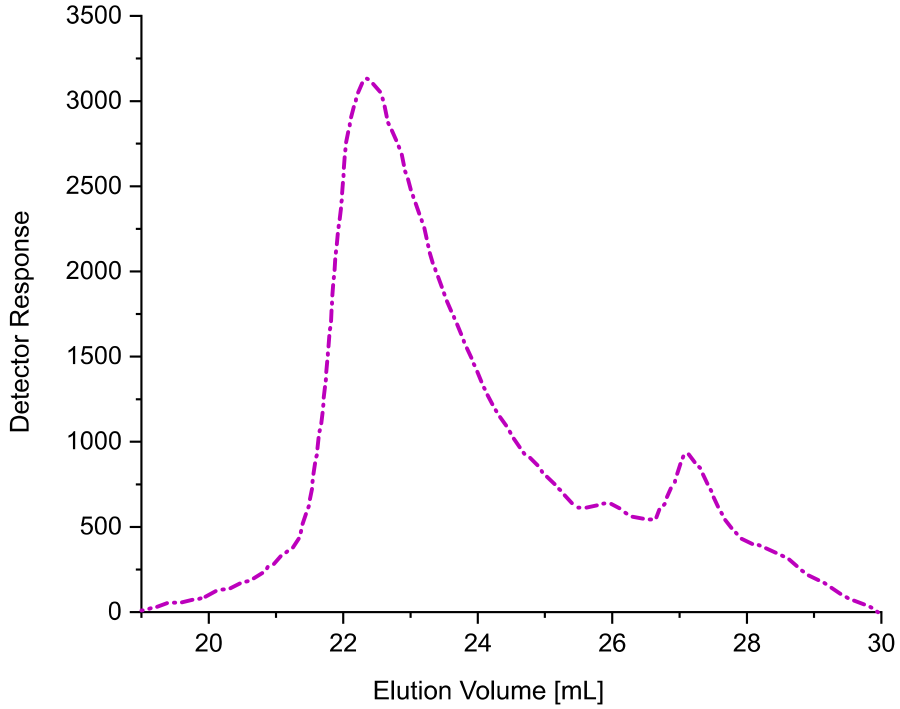

Figure S3. GPC chromatogram of Poly(Ani–AMPS), Mn=2,126 g/mol, Mw=9,345 g/mol, IPD=4.39.

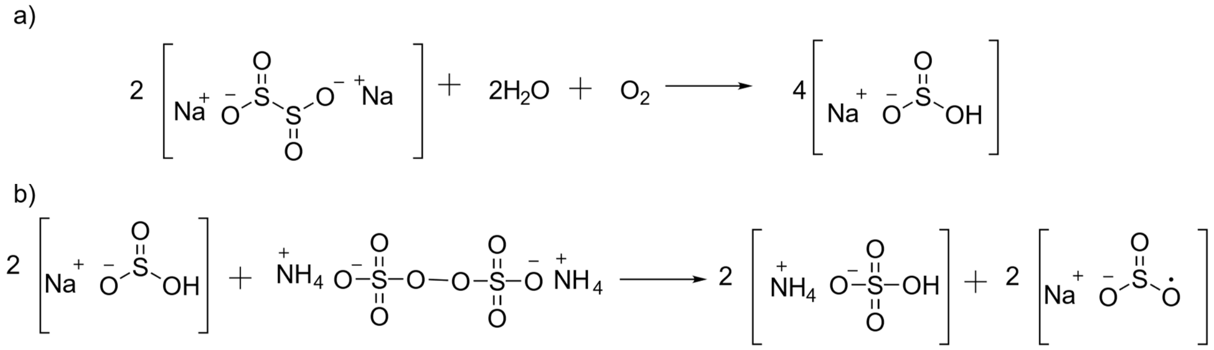

Figure S4. a) Scheme of the decomposition of sodium metabisulfite; b) its reaction with ammonium persulfate.

Table S1. Fluorescence intensity values concerning concentration of Fe^{2+}.

The intensity of emission signal at 348 nm in presence on Fe^{2+}					
Concentration of Fe^{2+} (μM)	1° Measurement	2° Measurement	3° Measurement	Mean	Standard Deviation

0	68.861	69.012	69.472	69.115	0.318
500	62.378	60.889	58.020	60.429	2.215
1000	46.135	46.507	46.206	46.283	0.197
1500	41.089	39.331	42.633	41.018	1.652
2000	35.700	34.506	35.042	35.083	0.598
2500	31.266	28.530	30.865	30.220	1.478
3000	25.885	25.493	27.207	26.195	0.898
3500	22.752	21.606	24.065	22.808	1.230
4000	19.955	18.954	18.681	19.197	0.671

Table S2. Fluorescence intensity values concerning concentration of Fe^{3+}.

The intensity of emission signal at 348 nm in presence on Fe^{3+}					
Concentration of Fe^{3+} (µM)	1° Measurement	2° Measurement	3° Measurement	Mean	Standard Deviation
0	68.861	69.012	69.472	69.115	0.318
20	57.426	57.825	59.055	58.102	0.849
40	52.886	51.003	51.870	51.920	0.942
60	45.470	44.002	45.338	44.937	0.812
80	39.535	40.946	38.248	39.576	1.349
100	36.006	35.730	34.203	35.313	0.971
150	29.931	30.645	29.580	30.052	0.543
200	25.693	25.424	24.287	25.135	0.746
250	19.899	19.788	20.625	20.104	0.455
300	14.358	15.337	14.814	14.836	0.490
350	10.262	11.210	10.889	10.787	0.482
400	7.501	7.072	7.048	7.207	0.255
